# Comparative transcriptome and WGCNA reveal key genes involved in lignocellulose degradation in *Sarcomyxa edulis*

**DOI:** 10.1038/s41598-022-23172-2

**Published:** 2022-11-01

**Authors:** Chao Duan, Feng-hua Tian, Lan Yao, Jian-Hua Lv, Chuan-Wen Jia, Chang-Tian Li

**Affiliations:** 1 Engineering Research Center of Chinese Ministry of Education for Edible and Medicinal Fungi, Jilin Agricultural University, Changchun, 130118 Jilin Province China; 2 Institute of Cotton Research, Shanxi Agricultural University, Yuncheng, 044000 Shanxi Province China; 3 Department of Plant Pathology, College of Agriculture, Guizhou University, Guiyang, China; 4 Institute of Edible Fungi, Guizhou University, Guiyang, China

**Keywords:** Genetics, Microbiology, Molecular biology

## Abstract

The developmental transcriptomes of *Sarcomyxa edulis* were assessed to explore the molecular mechanisms underlying lignocellulose degradation. Six stages were analyzed, spanning the entire developmental process: growth of mycelium until occupying half the bag (B1), mycelium under low-temperature stimulation after occupying the entire bag (B2), appearance of mycelium in primordia (B3), primordia (B4), mycelium at the harvest stage (B5), and mature fruiting body (B6). Samples from all six developmental stages were used for transcriptome sequencing, with three biological replicates for all experiments. A co-expression network of weighted genes associated with extracellular enzyme physiological traits was constructed using weighted gene co-expression network analysis (WGCNA). We obtained 19 gene co-expression modules significantly associated with lignocellulose degradation. In addition, 12 key genes and 8 kinds of TF families involved in lignocellulose degradation pathways were discovered from the four modules that exhibited the highest correlation with the target traits. These results provide new insights that advance our understanding of the molecular genetic mechanisms of lignocellulose degradation in *S. edulis* to facilitate its utilization by the edible mushroom industry.

## Introduction

*Sarcomyxa edulis* (Basidiomycota, Mycenaceae) degrades and utilizes forestry and agricultural residues rich in lignocellulose to support the development of its fruiting bodies^1^. When the environment is suitable, the mycelia absorb nutrients from substrates to produce fruiting bodies, which substantially affects the yield and quality of the edible mushrooms^2^. The capacity of *S. edulis* to degrade lignocellulose and the key genes and enzymes involved in its lignocellulose degradation pathways remain poorly studied. On the other hand, preliminary research on lignocellulose degradation has been conducted on model fungi, such as *Phanerochaete chrysosporium*^3^. Thus, a thorough investigation of lignocellulose degradation and utilization by *S. edulis* could provide valuable information benefiting the edible mushroom industry.

Due to the continuous improvements in high-throughput sequencing technologies and reductions in costs, transcriptome sequencing has been increasingly used in the biological sciences, dramatically improving the capacity and efficiency of gene discovery^4–6^. Traditional comparative analyses of small numbers of genes cannot handle massive amounts of data, which has led to the development of bioinformatics tools and analytical methods^7^. Weighted Gene co-expression Network Analysis (WGCNA) can be implemented to screen genes highly associated with target traits, obtain a series of biologically significant co-expression modules, and identify key genes underlying such traits^8^. WGCNA has been used in various fields in biology due to its accuracy and efficiency as a bioinformatics and biological data mining tool. WGCNA has played an important role in the analysis of multiple transcriptome data sets in many plants, such as *Brassica rapa*^9^ and strawberry^10^, and fungi, such as Chinese cordyceps^11^.

In this study, we analyzed the developmental transcriptomes of artificially cultivated *S. edulis* at six developmental stages. We implemented WGCNA to identify modules of co-expressed genes and key candidate genes in each developmental stage. We focused on the key genes involved in lignocellulose degradation pathways and identified four modules exhibiting high correlations with target traits. Our results provide novel insights into the molecular networks underlying *S. edulis* development, further elucidating the molecular genetic mechanisms of lignocellulose degradation.

## Materials and methods

### Collection of fungal samples at different developmental stages

The *Sarcomyxa edulis* strain (No. 2016120327) was used in this study. It is maintained in the Chinese Ministry of Education, Engineering Research Center for Edible and Medicinal Fungi, Jilin Agricultural University, China. Polypropylene bags were used as containers for the cultivation substrates and contained 1200 g medium, including 29.64% oak sawdust, 7.6% wheat bran, 0.38% gypsum, and 0.38% lime.

Sampling was carried out using tweezers to clamp 10 g of mycelium (together with culture medium) in the cultivation bags. The mycelia were then placed into a 20 ml sterile centrifuge tube. The samples were collected during the following growth stages: growth of mycelium until occupying half the bag (B1), mycelium under low-temperature stimulation after occupying the entire bag (B2), appearance of mycelium in primordia (B3), primordia (B4), mycelium at the harvest stage (B5), and mature fruiting body (B6). Three samples were taken each time from each developmental stage, mixed well, and then immediately stored at − 80 °C.

### RNA isolation, library construction, and sequencing

Total RNA was extracted from the samples using the RNA prep Pure PlantPlus Kit (Tiangen Biotech, Beijing, China). RNA degradation and contamination were monitored on 1% agarose gels, and RNA purity and concentration were measured using the BIOSPEC-NANO® (230 V) spectrophotometer (SHIMADZU). Three biological replicates were used for the developmental stage sampled. Eighteen libraries were sequenced using Illumina HiSeq 4000 by Gene Denovo Biotechnology Co. (Guangzhou, China).

### Sequencing data analysis

High-quality clean reads were obtained after filtering out the raw reads containing adapters or low-quality bases by fastp^12^. The steps of reads filtering are as follows:

1. Remove the reads with adapter.
2. Remove the reads containing more than 10% N.
3. Remove all A-base reads.
4. Remove low-quality reads (Q ≤ 20).

After the rRNA reads of each sample are removed by bowtie2^13^, the remaining fragments mapped to the reference genome by HISAT2^14^. The reference genome sequence data was uploaded to the NCBI database (Genome accession number: QUOL00000000.1)^1^. Transcripts reconstruction was performed using Stringtie^15^. Gene expression levels was quantified by the RSEM^16^. The gene numbers and their expression levels s were analyzed by calculating the fragments per kilobase of each transcript per million mapped reads (FPKM) values. Genes with a fold change ≥ 2 and a false discovery rate (FDR) < 0.05 in comparison were denoted as significant DEGs. DEGs were then subjected to GO (http://www.geneontology.org/) and KEGG databases (https://www.kegg.jp/) enrichment analysis^17^.

### Analysis of key co-expression network genes

The filtered data were used for network construction and module identification. The lignocellulose degradation genes in *S. edulis* were combined with data on physiological and biochemical indexes from four mycelium growth periods (Suppl. Table S1). WGCNA analysis was performed using the OmicShare tools, a free online platform for data analysis (https://www.omicshare.com/tools). The key genes were identified through WGCNA analysis and gene-phenotype association analysis.

### Validation of DEGs by qRT-PCR

The expression levels of 8 genes, which showed significant changes in the transcriptional expression data of the different developmental stages, were screened by qRT-PCR analysis. Gene-specific primers were designed using Primer v5.0 (Suppl. Table S5). The 18S gene was used as an internal control for gene expression normalization^18,19^. The relative gene expression data were obtained from the Ct values using the 2^−ΔΔCt^ method^11^.

## Results

### Global transcriptomic analysis

**Figure 1:**
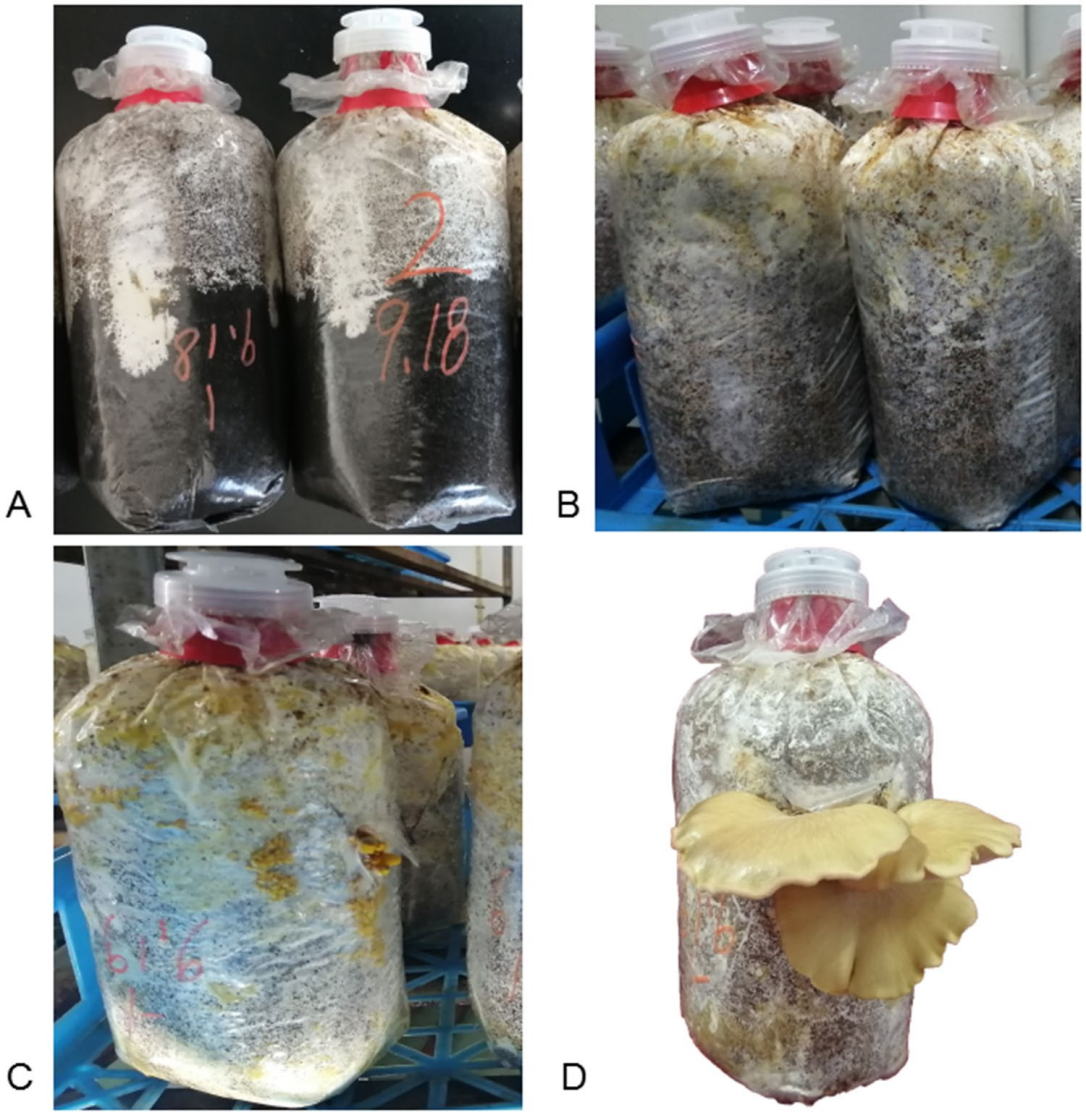
Developmental stages of *Sarcomyxa edulis*. (**A**) Mycelium growing to half bag. (**B**) Mcelium in cold stimulation after full bag. (**C**) Mycelium in primordia appearing and primordia, (**D**) Mycelium at the harvest stage and mature fruiting body.

To characterize gene expression patterns during development, 18 libraries were constructed using samples from the six developmental stages of *S. edulis* (Fig. 1). A total of 774.59 million raw reads were generated by Illumina paired-end sequencing. Which is 150 base pair by paired end sequenced. After cleaning and quality checks, 742.29 million clean reads were obtained, averaging 41.23 million reads per replicate (Suppl. Table S1). More than 77.56% of the reads per replicate could be mapped to the *S. edulis* genome. The Q_30_ percentage of all sequences in the 18 libraries was over 91%.

### Gene expression analysis

**Figure 2:**
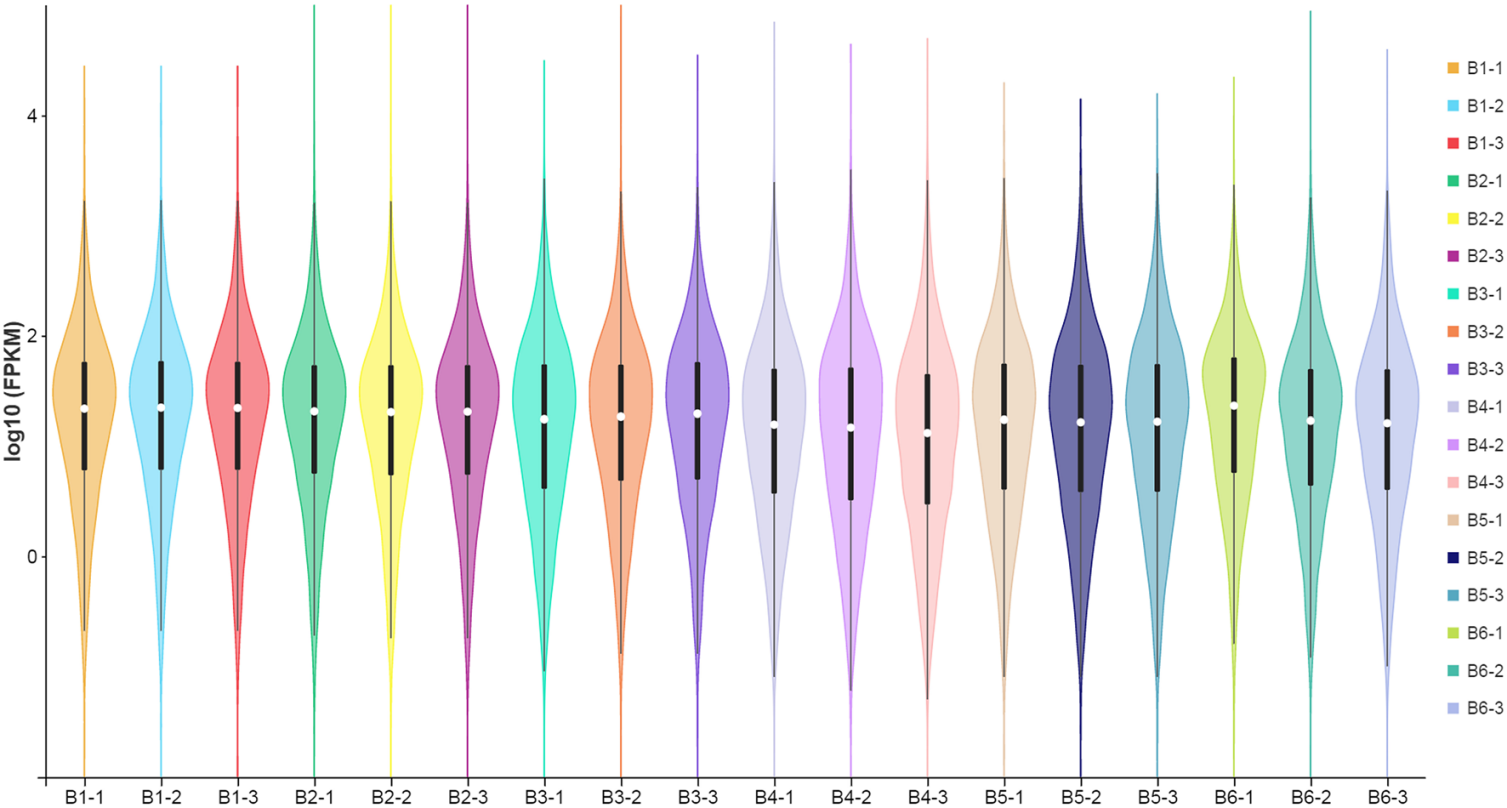
Violin map of horizontal distribution of gene expression (FPKM) in different samples. The abscissa is the name of the sample, and the ordinate is log10 (FPKM). The values from top to bottom represent the maximum, the upper quartile, the median, the lower quartile and the minimum in turn. The width of each violin represents the number of genes under the same expression.

Values of FPKM greater than 1 indicate that the gene is expressed, and higher FPKM values indicate higher expression. The gene expression analysis results revealed that the median of the 18 samples was similar in values, and the log_10_ FPKM values ranged from 3.82 to 4.59. The gene expression levels were highest in the B2 stage (log_10_ FPKM, 16.13) (Fig. 2; Suppl. Table S2).

### Identification of differentially expressed genes across various developmental stages

**Figure 3:**
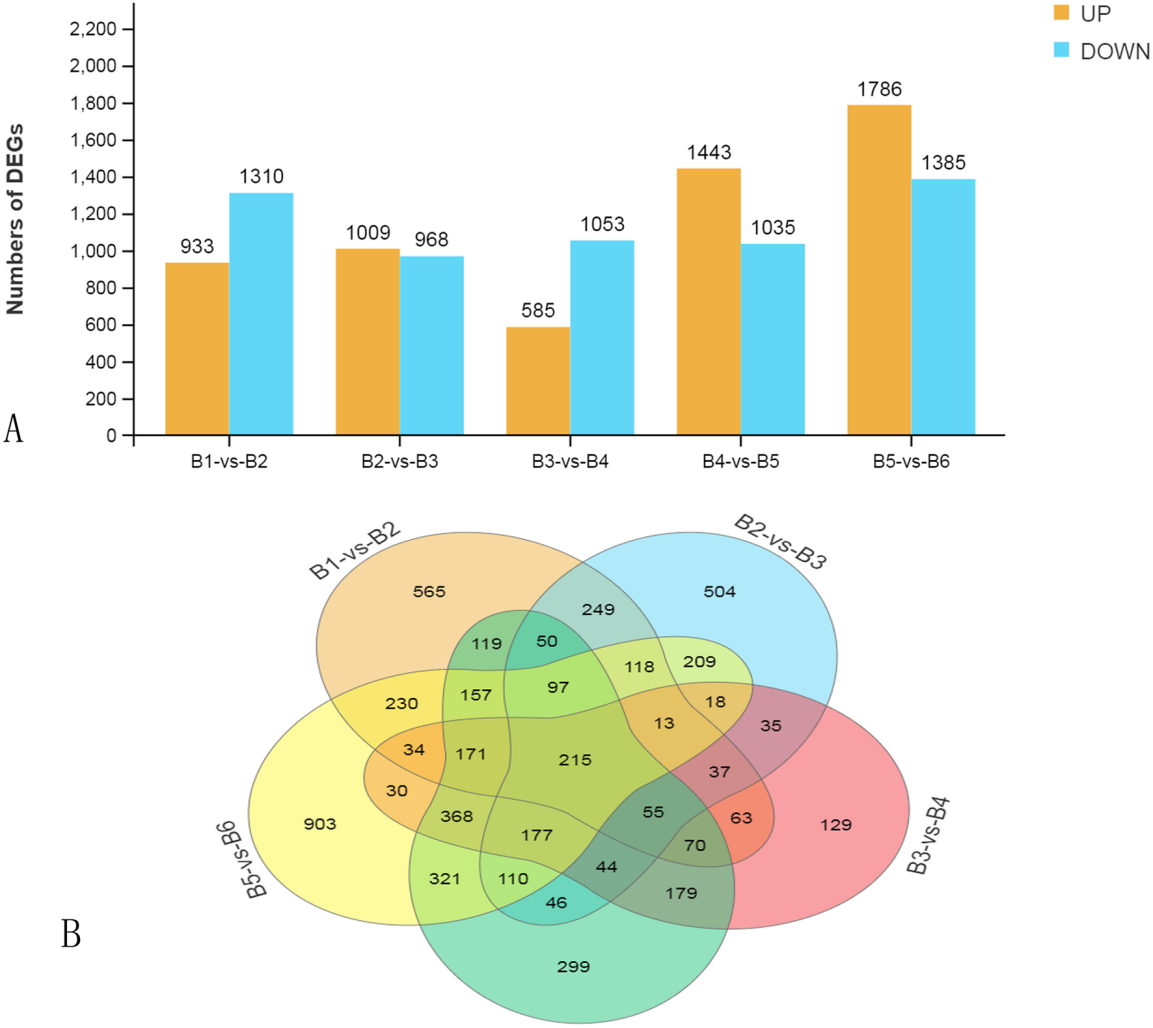
Differentially expressed genes across various developmental stages. (**A**) Histogram of differentially expressed genes. (**B**) Venn diagrams of differentially up-regulated genes.

The highest number of DEGs was observed between the B5 and B6 stages (3,171), followed by B4 versus B5 (2,478) and B1 versus B2 (2,243) (Fig. 3A). These results indicated that the expression profiles in B4 and B6 differed considerably compared to B5. The greatest number of unique DEGs were observed in the B5 versus B6 comparison (903). On the other hand, only 153 DEGs were unique to the B3 versus B4 comparison (Fig. 3B). There were 215 shared DEGs among all five performed comparisons between the six stages. These were primarily enriched in the GO category response to catalytic activity (GO:0003824) (Suppl. Fig. S2).

### Functional classification of the differentially expressed genes

All DEGs were classified into three categories: biological process (BP), cellular component (CC), and molecular function (MF). The significantly enriched terms of the DEGs in the B1 vs. B2, B2 versus B3, B3 versus B4, B4 versus B5, and B5 versus B6 comparisons were highly similar (Suppl. Fig. S3A–E).

The DEGs were mapped to the Kyoto Encyclopedia of Genes and Genomes (KEGG) database to evaluate their functions. The significantly enriched pathways of the DEGs in the B1 versus B2, B2 versus B3, B3 versus B4, B4 versus B5, and B5 versus B6 comparisons shared high similarity. All the significantly enriched pathways were assigned to “global and overview maps” and “carbohydrate metabolism” within the “Metabolism” pathways (Suppl. Table S3).

### Gene co-expression networks construction

The minimum power value was used in subsequent analyses when the correlation coefficients reached their plateau values (or values greater than 0.8; as shown on the left of Suppl. Fig. S4). We determined the changes in average gene connectivity under different power values (as shown on the right of Suppl. Fig. S4). The minimum power value of 8 was used for the following analysis.

**Figure 4:**
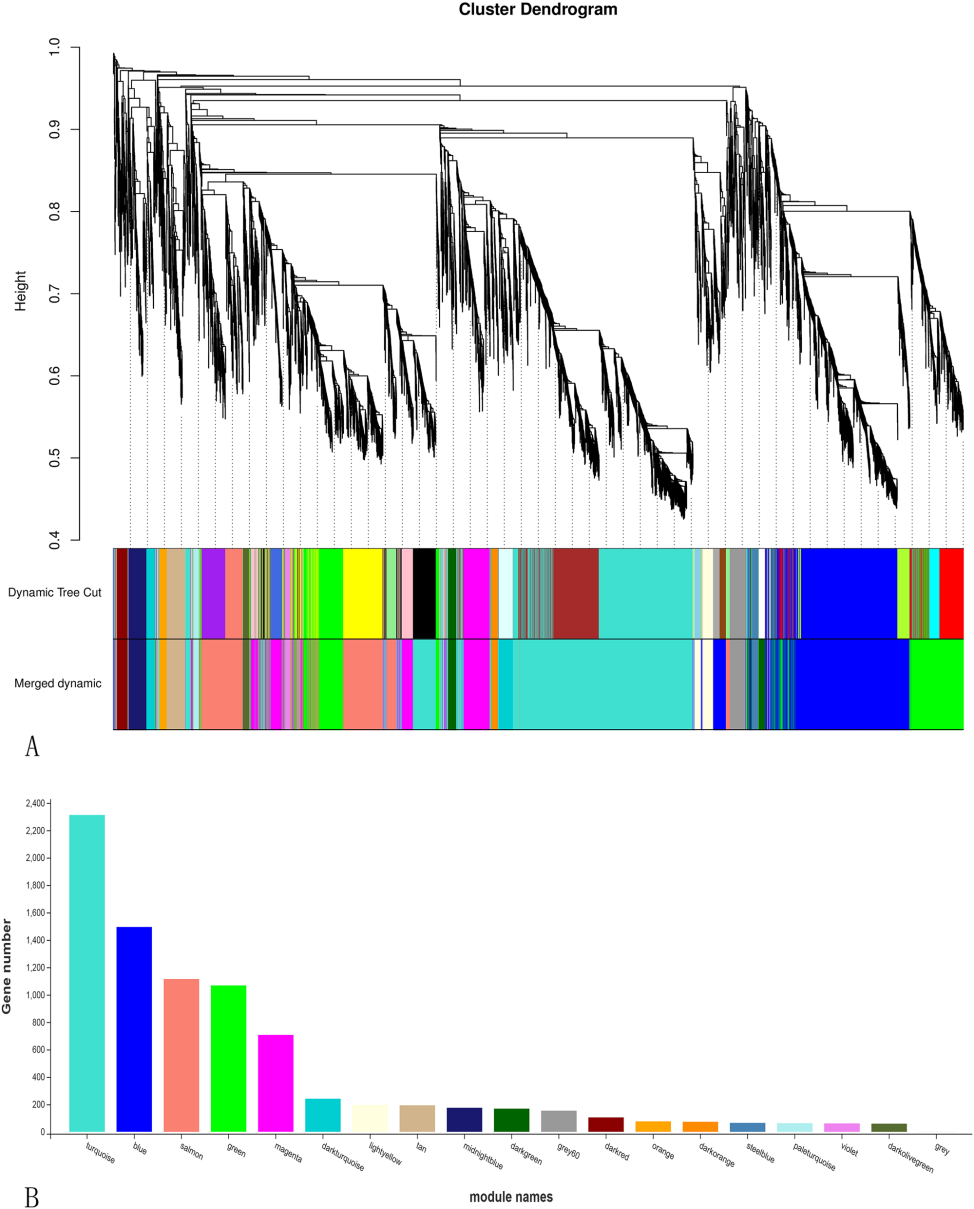
Nineteen different modules identified. (**A**) Gene co-expression network gene clustering number and modular cutting. Dynamic Tree Cut is the module divided according to clustering results. Merged Dynamic is the module division of merged modules with similar expression patterns according to module similarity. The subsequent analysis is conducted in accordance with merged modules. In the case of trees, the vertical distance represents the distance between two nodes (between genes), and the horizontal distance is meaningless. (**B**) Number of genes per module. The abscissa represents each module, and the ordinate represents the number of genes.

The 19 modules (marked with different colors, Fig. 4A), each corresponding to a branch of the gene clustering tree, were analyzed. The 19 modules correlated with different stages of development, indicating that the expression profiles were developmental stage-specific. The number of genes in each module is shown in Fig. 4B. The highest number of genes (2,312) was observed in the turquoise module, while the lowest (1 gene) was observed in the gray module.

**Figure 5:**
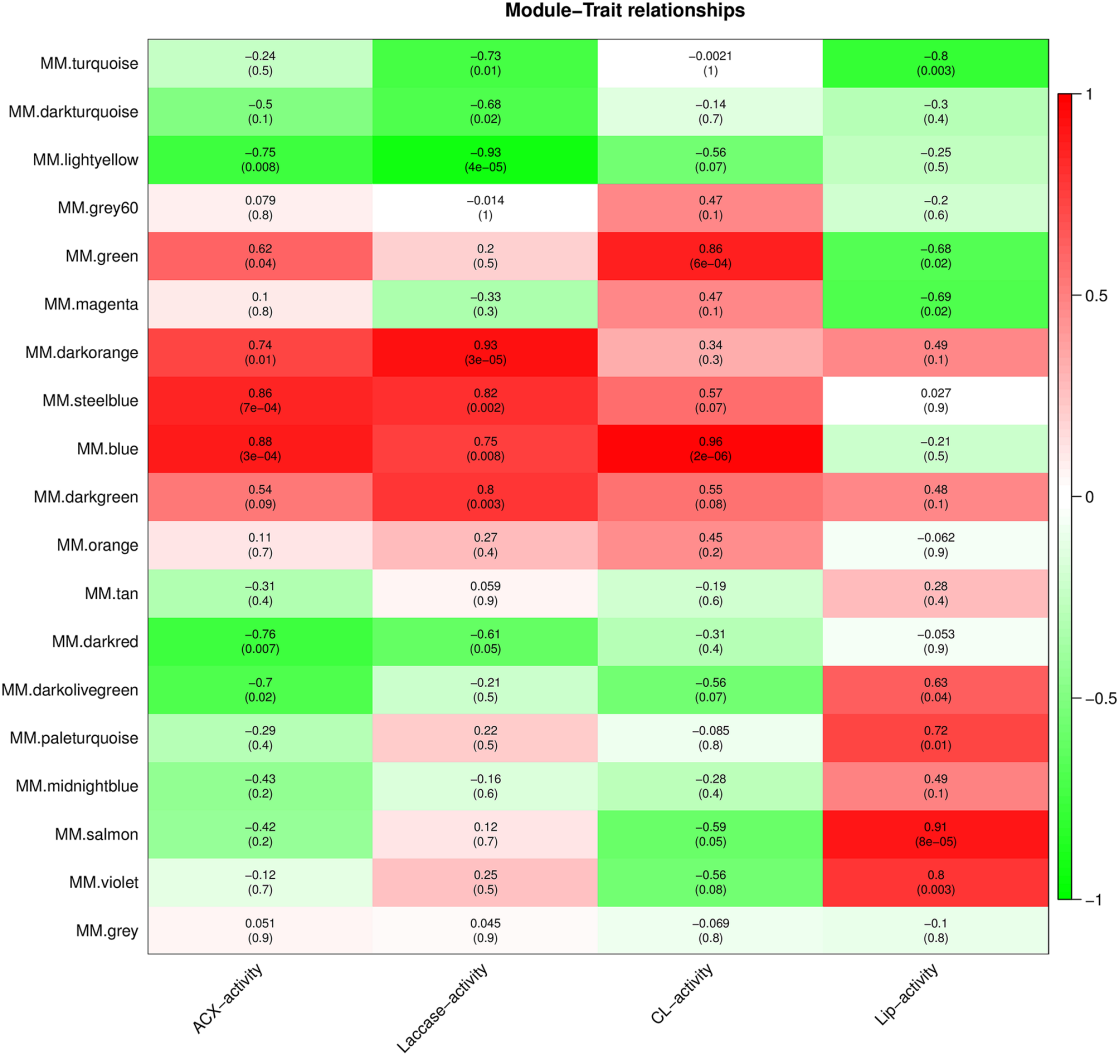
Association analysis of gene co-expression network modules with physiological and biochemical traits. The horizontal axis represents different characteristics, and the vertical axis represents each module. The red lattice represents a positive correlation between the physiological traits with the module, while the green lattice represents a negative correlation.

Correlation analyses between module eigenvalues and specific traits and phenotypic data were conducted to identify the modules with the highest potential associations with traits and phenotypes. Correlation analyses were conducted between four physiological and biochemical traits (laccase, acidic xylanase (ACX), cellulase (CL), and lignin peroxidase (Lip) activities) of the samples with the above modules under different developmental stages (Suppl. Table S4). Certain modules were highly correlated with physiological and biochemical traits (Fig. 5). For example, the blue module was significantly positively correlated with CL and ACX (r = 0.96, r = 0.88, respectively). Furthermore, a significant positive correlation was observed between the dark orange module and laccase (r = 0.93), laccase and ACX (r = 0.82, r = 0.86), the salmon module and Lip (r 128 = 0.91), and the salmon module and Lip (r = 0.91).The genes in these four modules were further evaluated.

### GO annotation of target modules

We mapped the genes in each module to the GO database (http://www.geneontology.org/), to further explore their function. We calculated the number of genes for each term to obtain the list of genes and statistics associated with the GO functions. The genes in these four modules were significantly enriched in several GO pathways in BP, MF, and CC (Suppl. Fig. S5). They were enriched in “catalytic activity” and “binding”, indicating that WGCNA effectively classified genes into biologically significant co-expression modules. These modules were the focus of our subsequent studies.

### Screening and functional analysis of key genes in target modules

The typical characteristic of a scale-free network is that most nodes in the network are only connected with a small number of nodes, and only a few are connected with most. Therefore, these nodes are the key nodes to identify, containing the so-called hub genes. These hub genes have a high degree of connectivity in their modules, making them biologically more meaningful than other nodes. The genes with MM value > 0.8 and *P* < 0.01 were screened from the above four modules as hub genes. Blue, darkgreen, lightcyan1 and cyan modules contained 675, 146, 175, and 131 hub genes, respectively. Then these hub genes were compared to the CAZY database (http://www.cazy.org, specific for carbohydrate enzyme genes)^20^, which further reduced the number of key genes to 64. Finally, combined with the annotation of NR, GO, and KEGG databases, a total of 12 key genes potentially related to lignocellulose degradation exhibiting the highest correlations with the target traits, were selected from the four modules (Table 1). 11 key genes were identified from the blue module, and 1 key gene were identified from the steelblue module.

**Table 1:** Core genes information table.

Module	Gene ID	Family in CAZy database	Description
Blue	SE.1A1237	AA9	Endoglucanase-7
Blue	SE.1A1239	AA9	Endoglucanase-7
Blue	SE.1A1422	AA3_2	Pyranose dehydrogenase 3
Blue	SE.1A1616	GH5_15	Endo-1,6-beta-glucosidase B
Blue	SE.1A1772	GH16_1	Endo-1,3(4)-beta-glucanase
Blue	SE.1A3039	GH5_9	Exo-1,3-beta-glucanase
Blue	SE.1A3347	GH5_9	Exo-beta-1,3-glucanase
Blue	SE.1A4339	GH16_1	Endo-beta-glucanase
Blue	SE.1A5866	AA2	Manganese peroxidase 1
Blue	SE.1A8947	AA1_1	Laccase-2
Blue	SE.1A9306	AA1_1	Laccase
Steelblue	SE.1A8861	GH51	Alpha-L-arabinofuranosidase

The 12 key genes are shown in Table 1. They were all annotated to the CAZy database, indicating that they all encode carbohydrate enzymes. This further confirmed the robustness of our analysis. Six genes belonged to the AA (auxiliary activity) family, six to the GH (glycoside hydrolase) family. one to the CBMs (Carbohydrate-Binding Modules) family, and one to the PLs (Polysaccharide Lyases) family. Among those, five endoglucanases and two exoglucanases were identified, which are cellulose-degrading enzymes^21^. There was one manganese peroxidase and two laccases, which are lignin-degrading enzymes^22^. One pyranose dehydrogenase was identified, which functions as an auxiliary enzyme of a lignin-degrading enzyme^23^. There was also one arabinofuranase that is involved in hemicellulose degradation^24^.

### Differential expression of lignocellulose degradation-related genes

**Figure 6:**
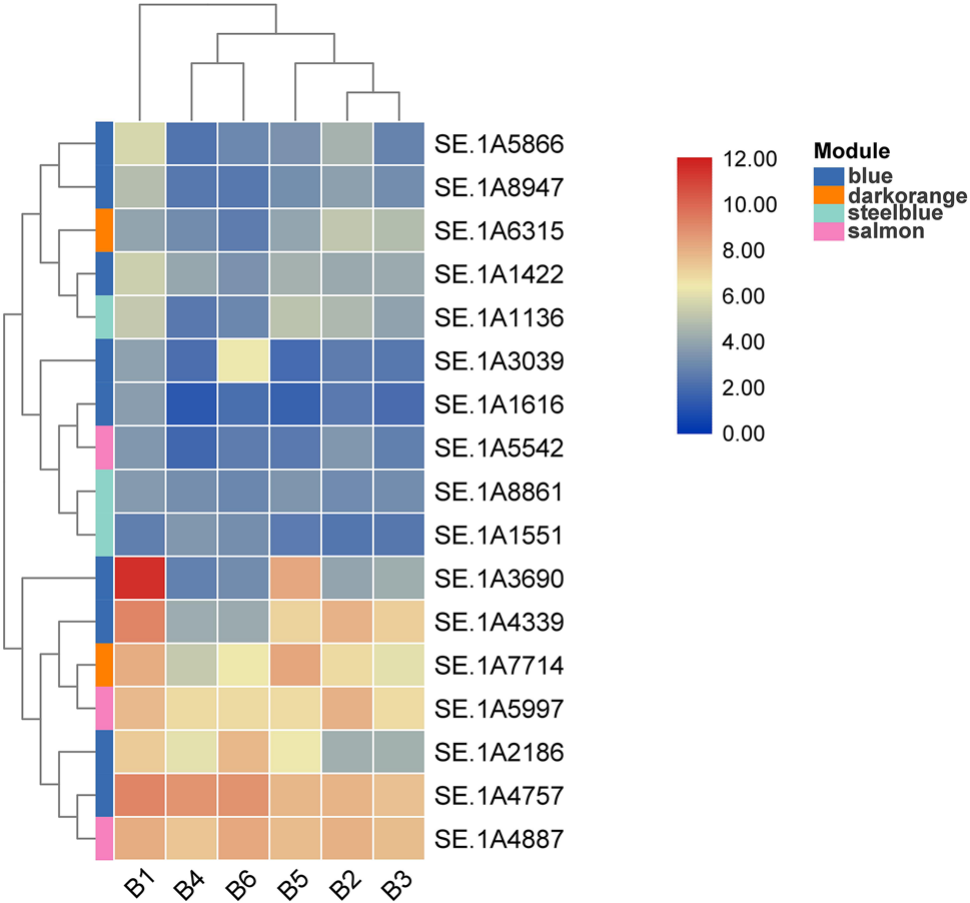
Heatmap clustering of key genes in the six stages. The levels of expression are represented by log2 (FPKM) values after centralization correction. Genes with similar patterns of expression are clustered together.

The differential expression of 12 genes described above was analyzed by TBtools (Version: 0.674) (https://github.com/CJ-Chen/TBtools/releases)^25^ (Fig. 6). SE.1A3347 and SE.1A4339 were clustered together and exhibited high expression levels in all six stages. SE.A1616, SE.1A1237, SE.1A8861, and SE.1A9306 were clustered together and exhibited low expression. When comparing the six developmental stages, most genes were relatively highly expressed in A1, indicating that they were significantly correlated with lignocellulose degradation. SE.1A8947 and SE.1A9306 are both laccase enzymes clustered together, indicating that they exercise their biological functions together.

### Screening and functional analysis of transcription factors (TFs) in target modules

Using the filtering method described in “Screening and functional analysis of key genes in target modules” section, combined with the plant transcription factor database (http://planttfdb.gao-lab.org/), a total of 37 TFs potentially related to lignocellulose degradation were identified from the four modules showing the highest correlations with the target traits (Table 2). They belong to eight types of TF families, namely bHLH (basic helix loop helix dimerization region), bZIP (basic leucine zipper), C_2_H_2_ (zinc finger sequence contains two cysteines and two histidines), C_3_H (Cys_3_His zinc finger domain), GATA (proteins that interact with conserved WGATAR motifs), HB (homeo box), MYB (v-myb avian myeloblastosis viral oncogene homolog) and NF-YB (nuclear factor-YB). The bZIP family was the most populous, with 10 transcription factors, and the HB family the least, with 2 transcription factor.

**Table 2:** Core TFs information table.

Module	Description	Gene IDs
Blue	bHLH	SE.1A5173,SE.1A6394
	bZIP	SE.1A5531,SE.1A7482
	C_2_H_2_	SE.1A6490,SE.1A9236
	GATA	MSTRG.6731,SE.1A4109
	HB	SE.1A3375
	MYB	SE.1A4985,SE.1A7224
	NF-YB	SE.1A3518,SE.1A4952
Salmon	bHLH	SE.1A8084
	bZIP	MSTRG.2213,SE.1A0840,SE.1A3285.SE.1A3646, SE.1A5219,SE.1A5511,SE.1A6147,SE.1A6224
	C_2_H_2_	SE.1A2217,SE.1A3360,SE.1A5655,SE.1A6056, SE.1A7636,SE.1A9051
	C_3_H	SE.1A0661,SE.1A3307,SE.1A3970,SE.1A5510
	GATA	MSTRG.5813
	HB	MSTRG.7129
	MYB	SE.1A0338,SE.1A2973,SE.1A5093

### Validation of DEGs by quantitative real-time PCR (qRT-PCR)

To verify the reliability of the transcriptome data, eight DEGs related to lignocellulose degradation were selected for qRT-PCR analysis (Suppl. Fig. S6). The expression patterns of the selected eight genes evaluated with qRT-PCR were consistent with the expression patterns during the six developmental stages. However, a few genes differed at certain stages. These disparities maybe caused by the methodological differences between transcriptome sequencing and qRT-PCR, which have been shown to have a certain degree of inconsistency (approximately 30–40%)^26^. Therefore, overall the results thus did not exceed the expected range of deviation, as the qRT-PCR results were consistent with the transcriptome data for most genes.

## Discussion

The degradation and utilization of lignocellulose are pivotal processes for the yield and quality of edible fungi. Current bioinformatics and data analysis methods can further study these correlations and the genes underlying them through the identification of co-expression modules. Thousands of genes with similar biological functions were assigned to individual modules. By comprehensively investigating the biological significance of the modules, the functions of genes in each module can be determined, which aids the analysis of the characteristics of populations with complex genetic backgrounds.

After obtaining a large amount of transcriptome and physiological data, the next challenge is evaluating and identifying their biological implications. Network analysis is widely used to mine genomic, transcriptomic, and metabonomic datasets. WGCNA was used to identify genes associated with and potentially regulating lignocellulose degradation. The genes related to the target traits were identified and classified into co-expression modules with high biological significance, which has been shown to be an effective data mining approach. ACX, laccase, CL, and Lip are extracellular enzymes directly involved in lignocellulose degradation, and their functions can be used as target traits in WGCNA. Therefore, we analyzed 19 co-expression modules associated with physiological traits by WGCNA, four of which exhibited a high correlation with these traits. Further network analysis revealed the key genes underlying these four modules. These results indicate that WGCNA can be used to classify genes into co-expression modules and pathways with biological significance, providing key information on potential biological functions that can aid future research.

Laccase and peroxidase are important extracellular lignin degrading enzymes^27,28^. Peroxidases include lignin peroxidase (LiP) and manganese peroxidase (MnP). LiP and MnP oxidize corresponding substrates to form intermediate cation free radicals through two consecutive single electrons^29^. In lignin, laccase oxidizes phenol to phenoxy radicals, which may lead to the cleavage of aryl-C^30^. Laccase can also oxidize non-phenol substrates in the presence of certain auxiliary substrates, such as ABTS^31^. In this experiment, the activity of LiP was very low (Suppl. Table S4), so no LiP gene was selected as the key gene. It may be related to the limitations of test and analysis methods.

These TFs play a major role in edible fungi growth and development. bHLH transcription factorsare highly conserved in fungi, regulating the spore production process, sexual and asexual reproduction process, and promoting sclerotia formation^32^. The bZIP transcription factor is essential for fungal growth and conidiation in *Fusarium pseudograminearum*^33^. C_2_H_2_ transcription factors are involved in fungi pathogenicity, inhibition of carbon catabolites, regulation of differentiation of fruiting bodies^34^. Fungal GATA factors regulate nitrogen metabolism, light induction, siderophore biosynthesis and mating-type switching^35^. HB are essential for fungal differentiation and secondary metabolism in *Aspergillus nidulans*^36^. MADS—box factors interact with other regulatory proteins in complexes which can activate or repress transcription^37^. MYB transcription factors are involved in cell differentiation, cycle regulation, secondary metabolism, and morphogenesis^38^. However, there is almost no research on their degradation of lignocellulose, and we will conduct in-depth research in the future.

## Supplementary Information

Supplementary Information 1.

Supplementary Information 2.

Supplementary Information 3.

Supplementary Information 4.

Supplementary Information 5.

Supplementary Information 6.

Supplementary Information 7.

Supplementary Information 8.

Supplementary Information 9.

Supplementary Information 10.

Supplementary Information 11.

## Data Availability

The raw data have been submitted under BioProject number PRJNA739377 to the Sequence Read Archive (SRA) database at NCBI (https://dataview.ncbi.nlm.nih.gov/object/PRJNA739377?reviewer=dlp50ehjesb7chmkqnl39ofhk6).
