# Supplementary figures and images for: Comparative transcriptome and WGCNA reveal key genes involved in lignocellulose degradation in Sarcomyxa edulis
Source: Sci Rep. 2022 Nov 1;12:18379. doi: 10.1038/s41598-022-23172-2 (PMC9626453; doi:10.1038/s41598-022-23172-2)

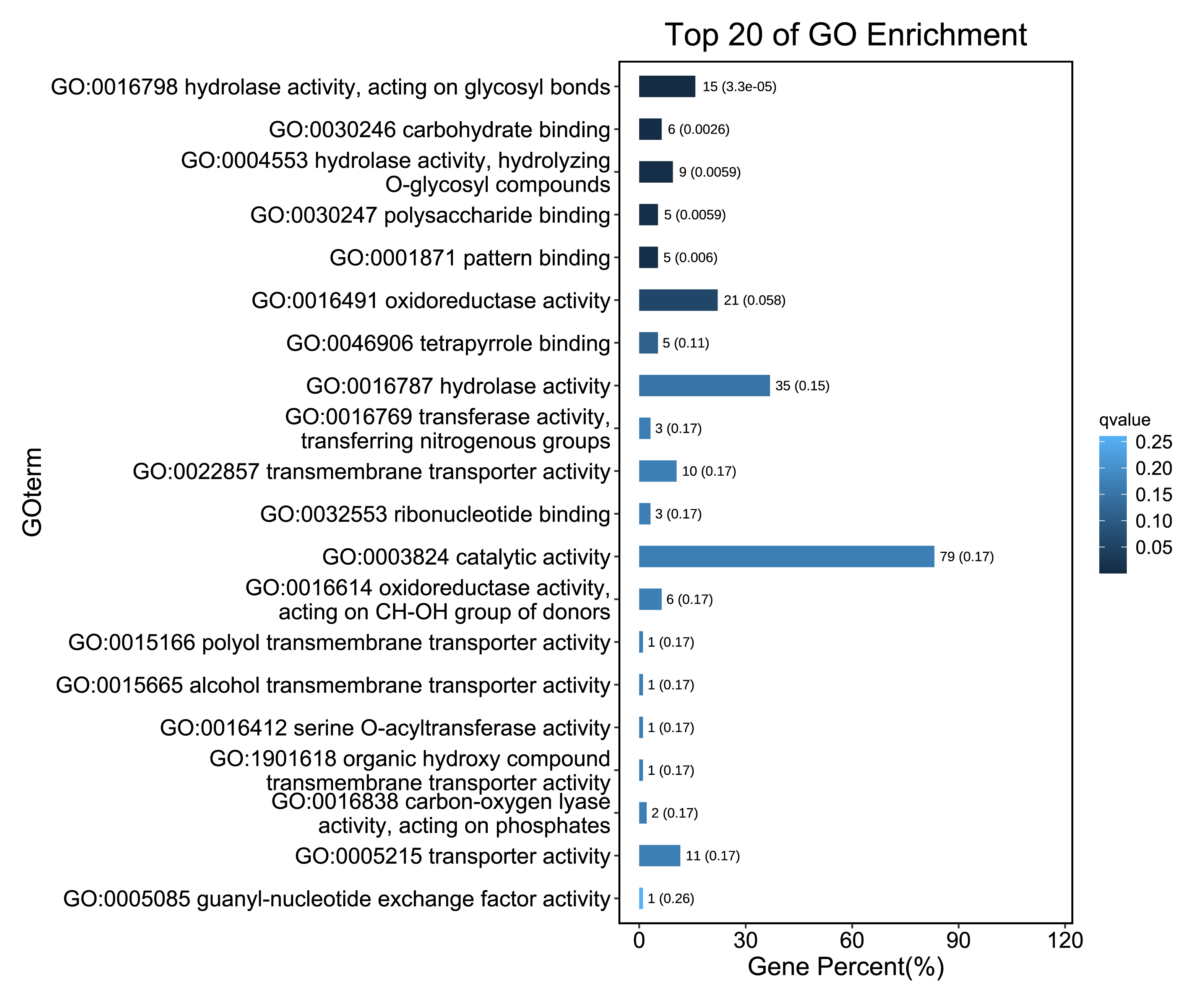


Fig. S1. Top 20 of GO Enrichment for 215 different genes.

Supplement: Supplementary file 1 — Supplementary Information 1. [file 41598_2022_23172_MOESM1_ESM.docx]

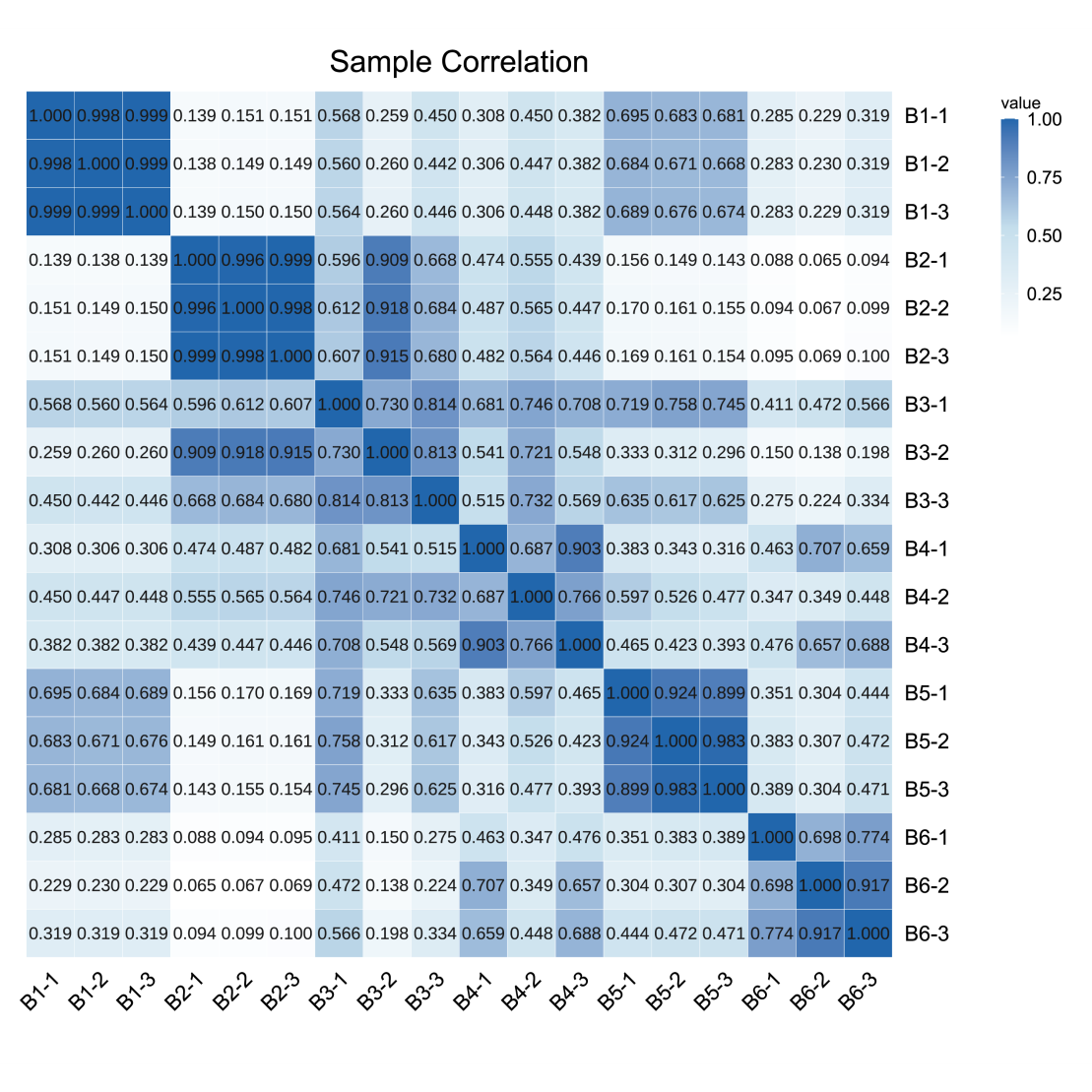


Fig. S2. Pearson correlation between samples.

Supplement: Supplementary file 2 — Supplementary Information 2. [file 41598_2022_23172_MOESM2_ESM.docx]
