# Supplementary material for: Comparative transcriptome and WGCNA reveal key genes involved in lignocellulose degradation in Sarcomyxa edulis
Source: Sci Rep. 2022 Nov 1;12:18379. doi: 10.1038/s41598-022-23172-2 (PMC9626453; doi:10.1038/s41598-022-23172-2)
Supplement: Supplementary file 3 — Supplementary Information 3. [file 41598_2022_23172_MOESM3_ESM.doc]

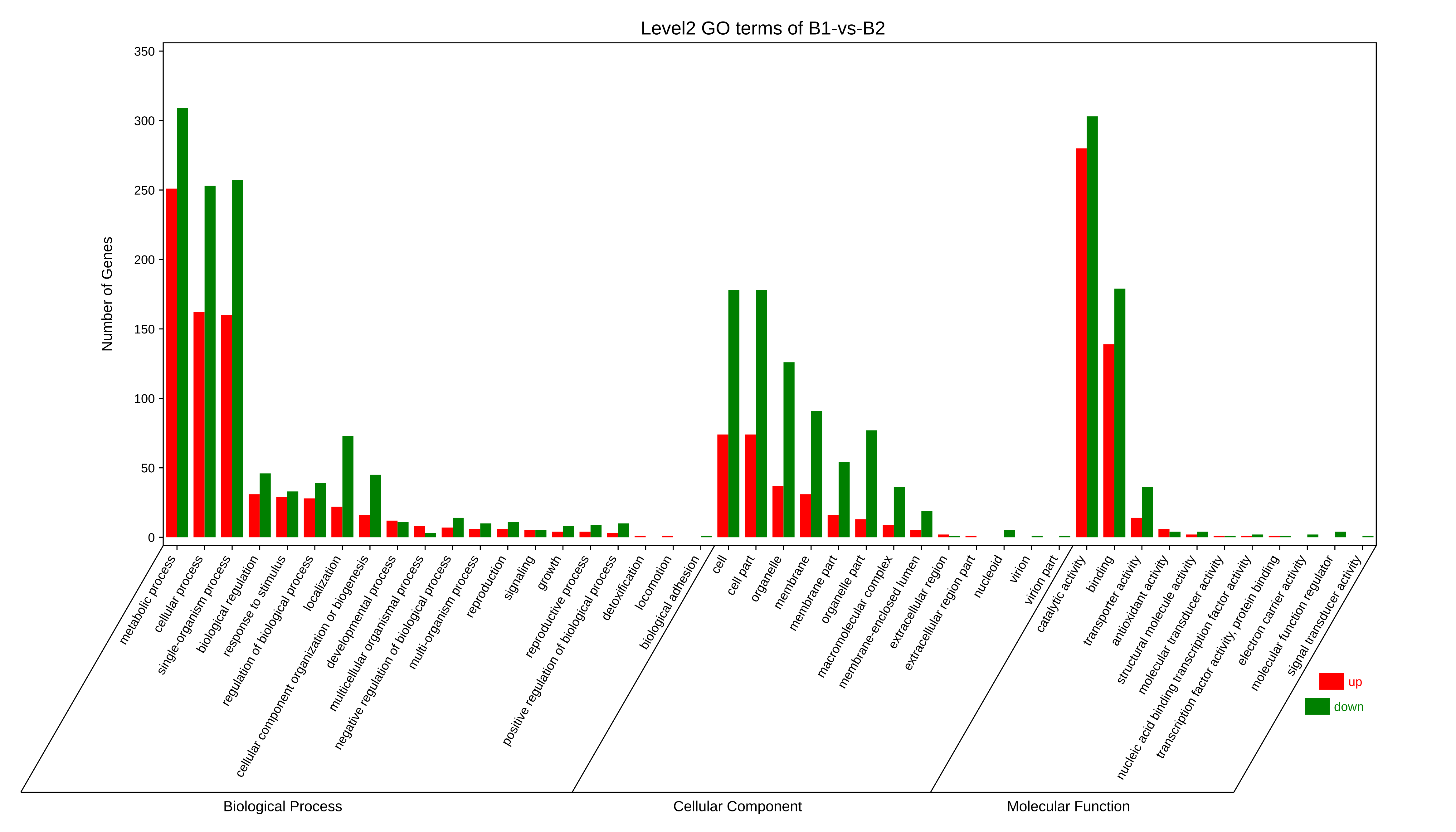


A: GO analysis of B1-vs-B2


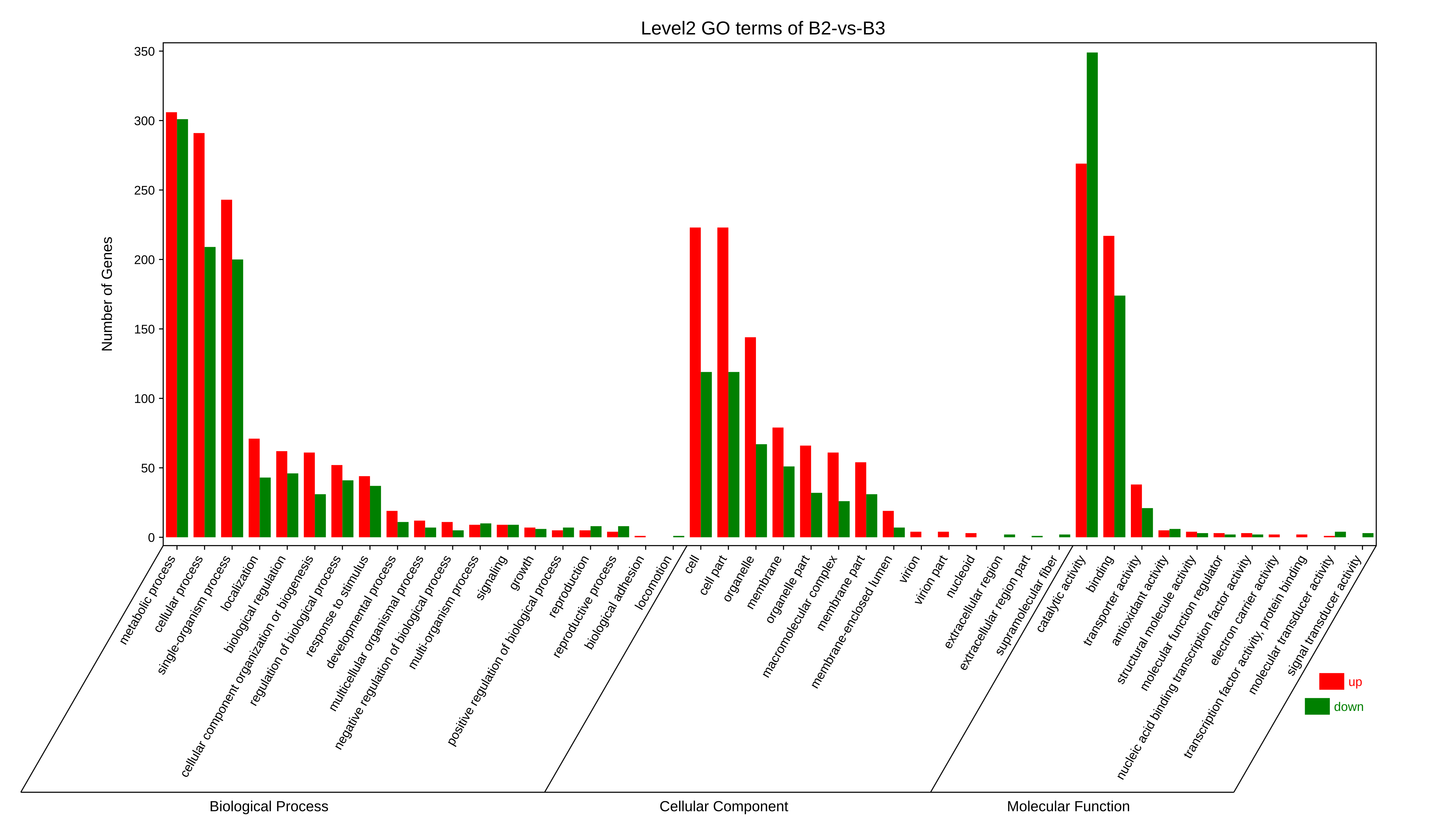


B: GO analysis of B2-vs-B3.


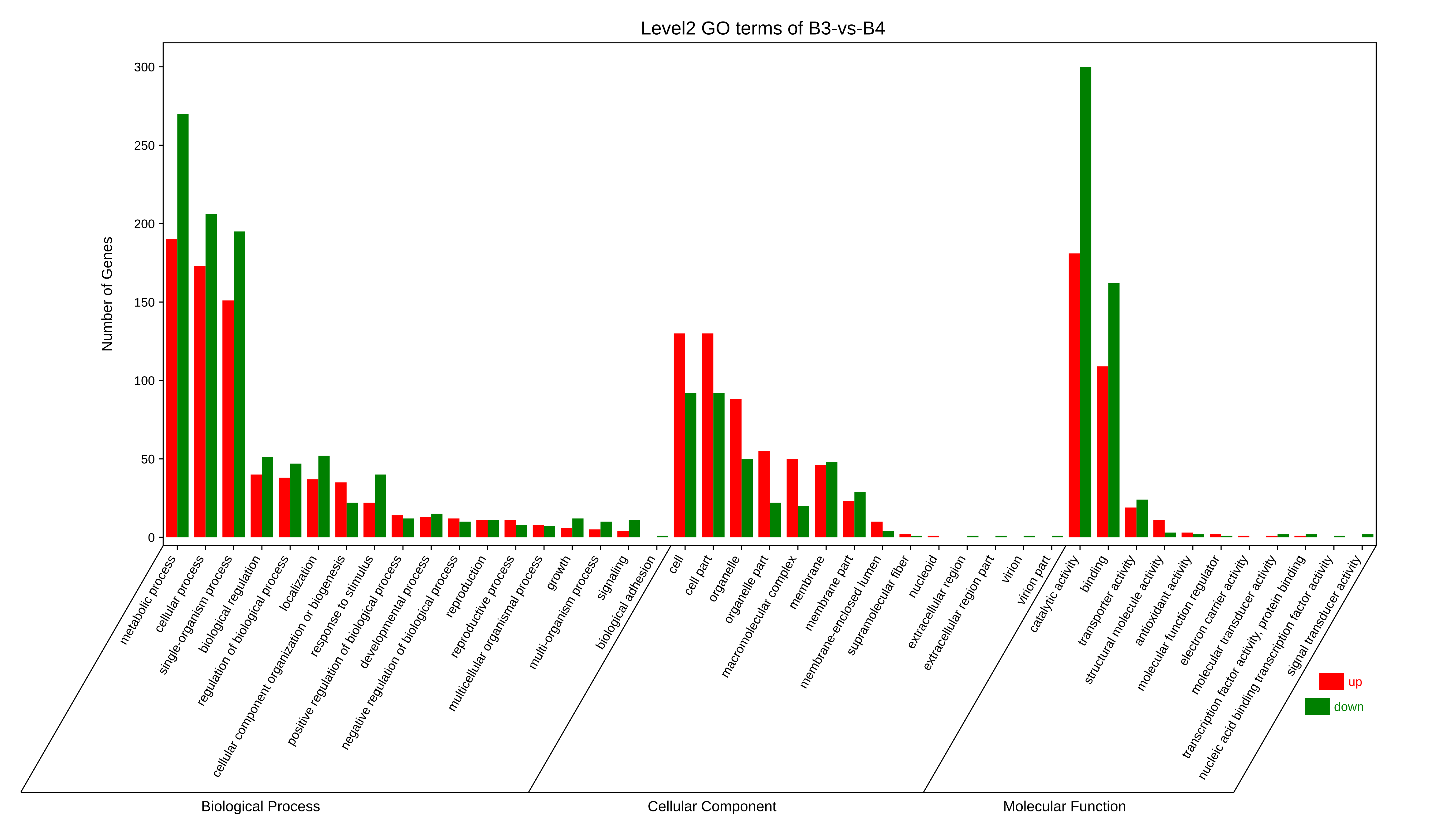


C: GO analysis of B3-vs-B4.


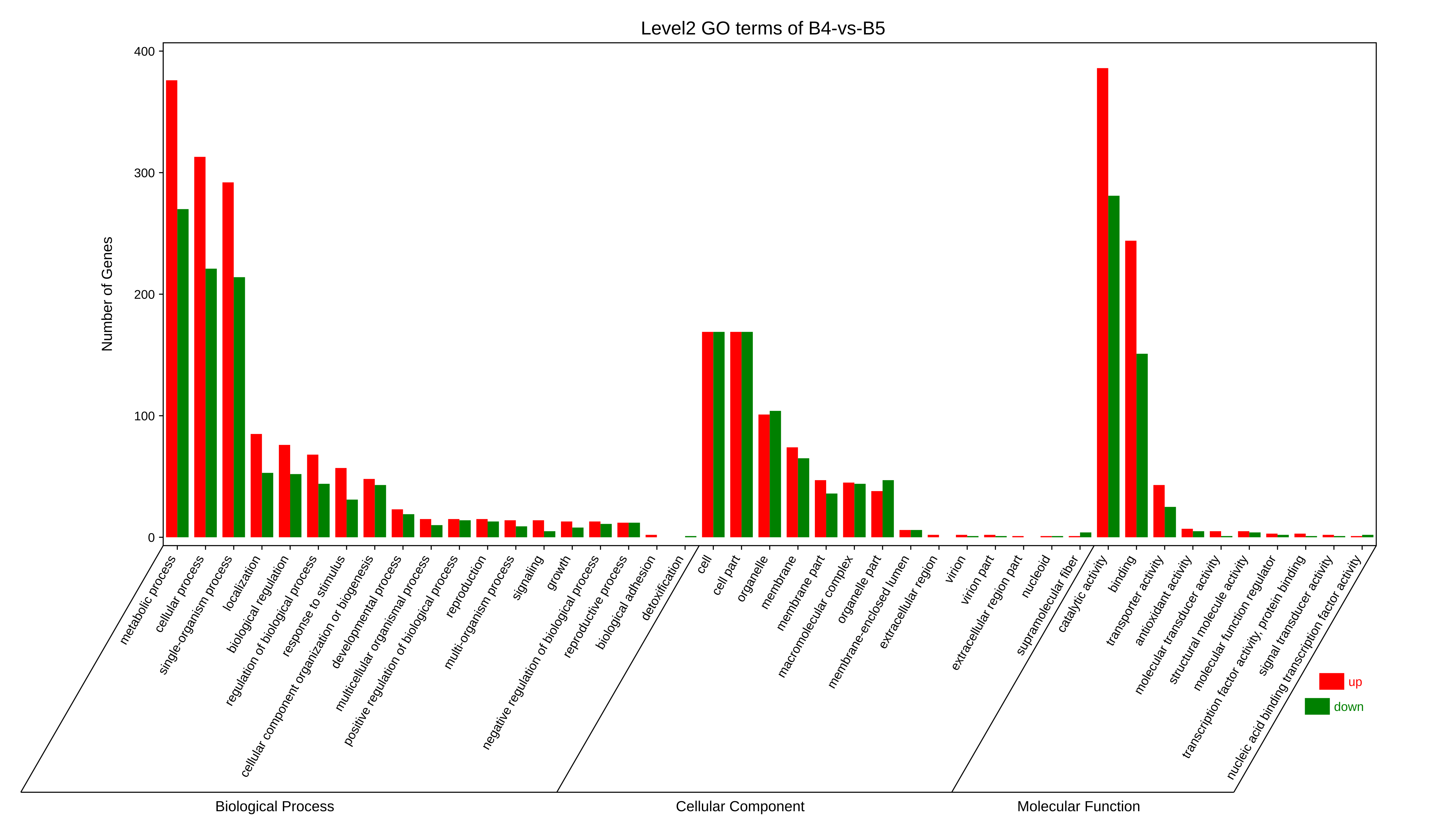


D: GO analysis of B4-VS-B5.


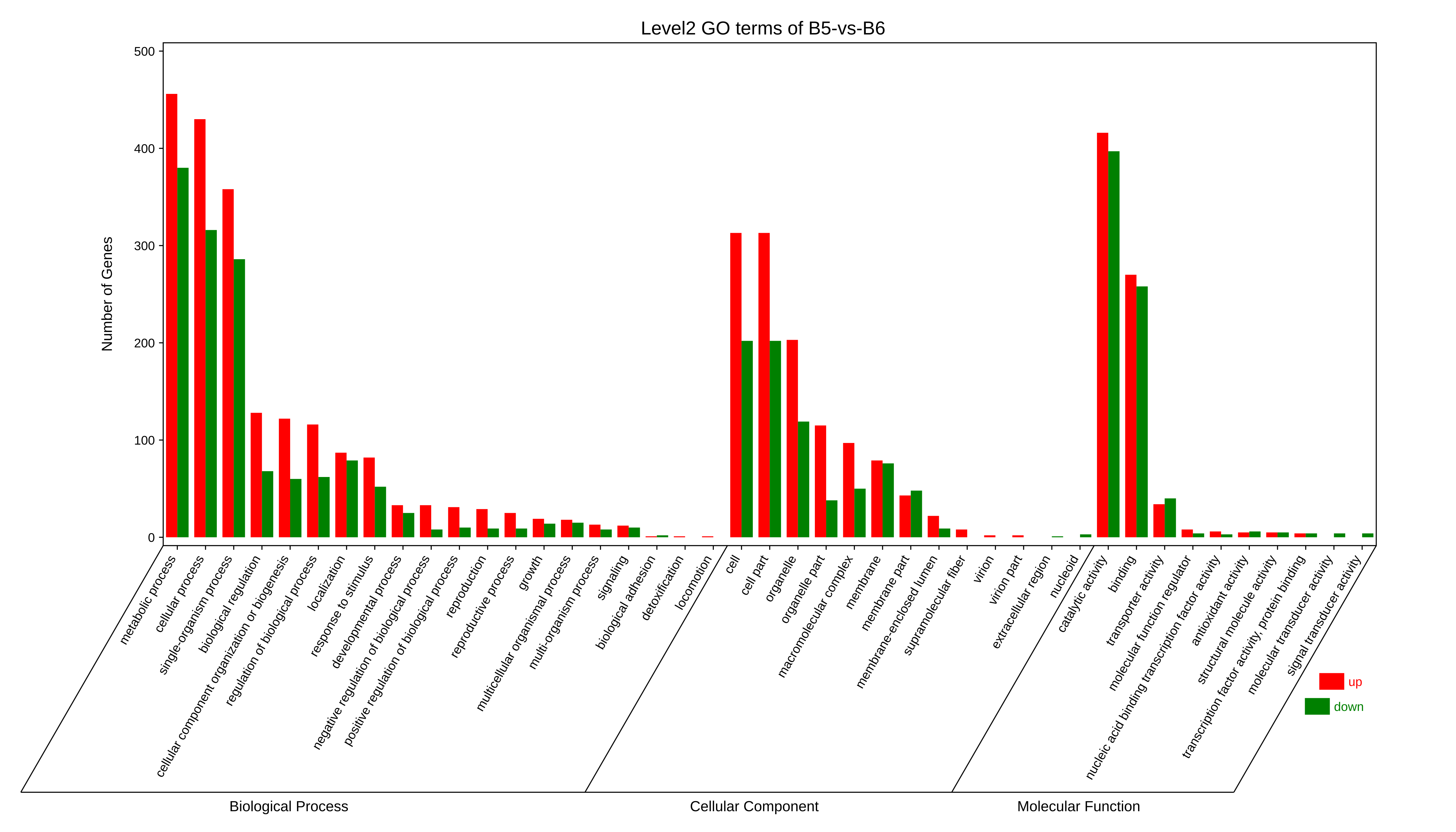


E: GO analysis of B5-VS-B6.

**Fig. S3.** GO functional classification of differentially expressed genes. The green bars represent biological processes; yellow bars represent cellular components; blue bars represent molecular functions. Only the significant Go terms (P< 0.005) were shown.
