# Supplementary material for: Comparative transcriptome and WGCNA reveal key genes involved in lignocellulose degradation in Sarcomyxa edulis
Source: Sci Rep. 2022 Nov 1;12:18379. doi: 10.1038/s41598-022-23172-2 (PMC9626453; doi:10.1038/s41598-022-23172-2)
Supplement: Supplementary file 4 — Supplementary Information 4. [file 41598_2022_23172_MOESM4_ESM.doc]

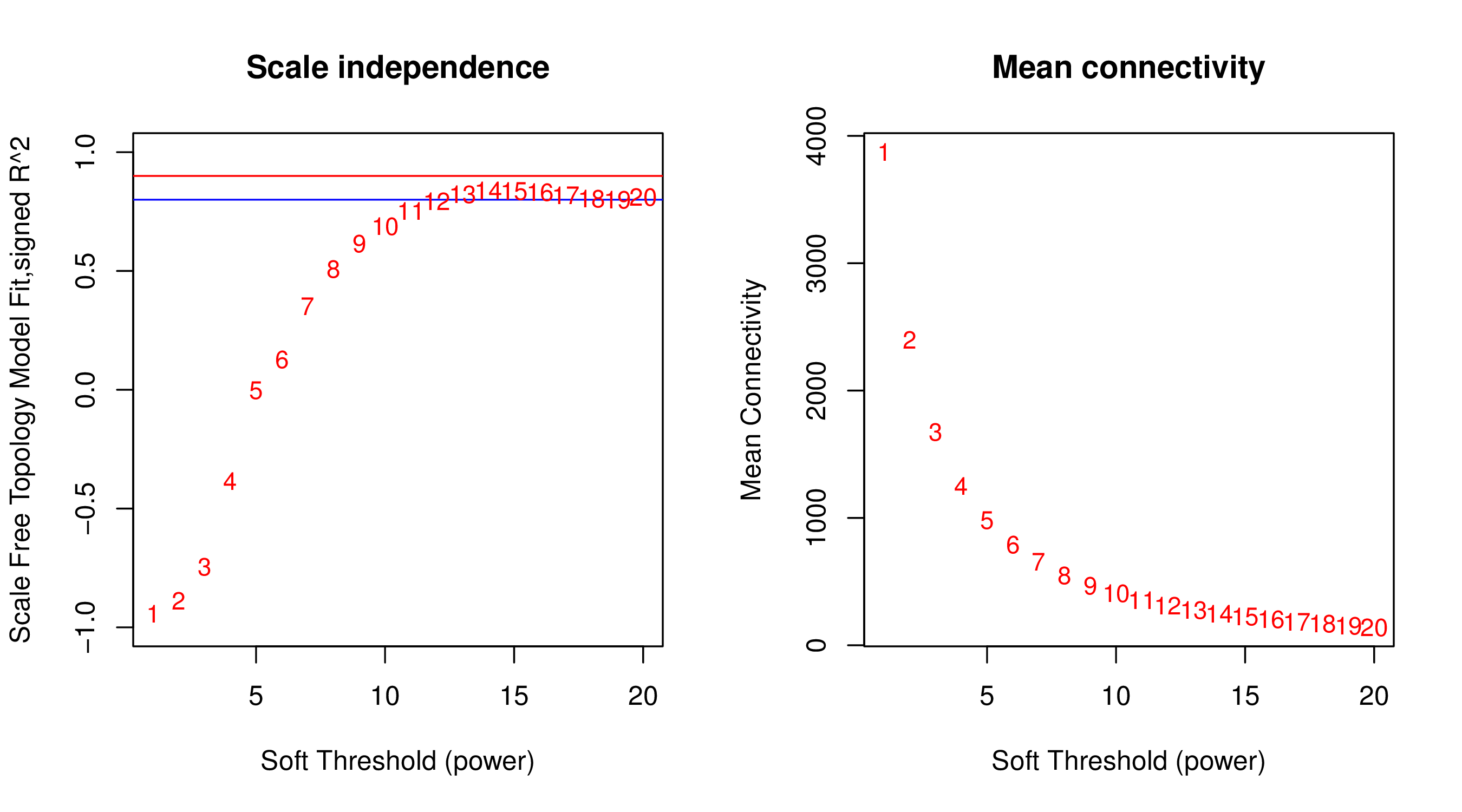


**Fig. S4.** Graph of Power value. Left: the abscissa represents the power value, the ordinate represents the correlation coefficient, the blue line represents the correlation coefficient of 0.8, and the red line represents the correlation coefficient of 0.9. Right: the abscissa represents the power value, and the ordinate represents the average connectivity of genes.
