# Supplementary material for: Comparative transcriptome and WGCNA reveal key genes involved in lignocellulose degradation in Sarcomyxa edulis
Source: Sci Rep. 2022 Nov 1;12:18379. doi: 10.1038/s41598-022-23172-2 (PMC9626453; doi:10.1038/s41598-022-23172-2)
Supplement: Supplementary file 5 — Supplementary Information 5. [file 41598_2022_23172_MOESM5_ESM.doc]

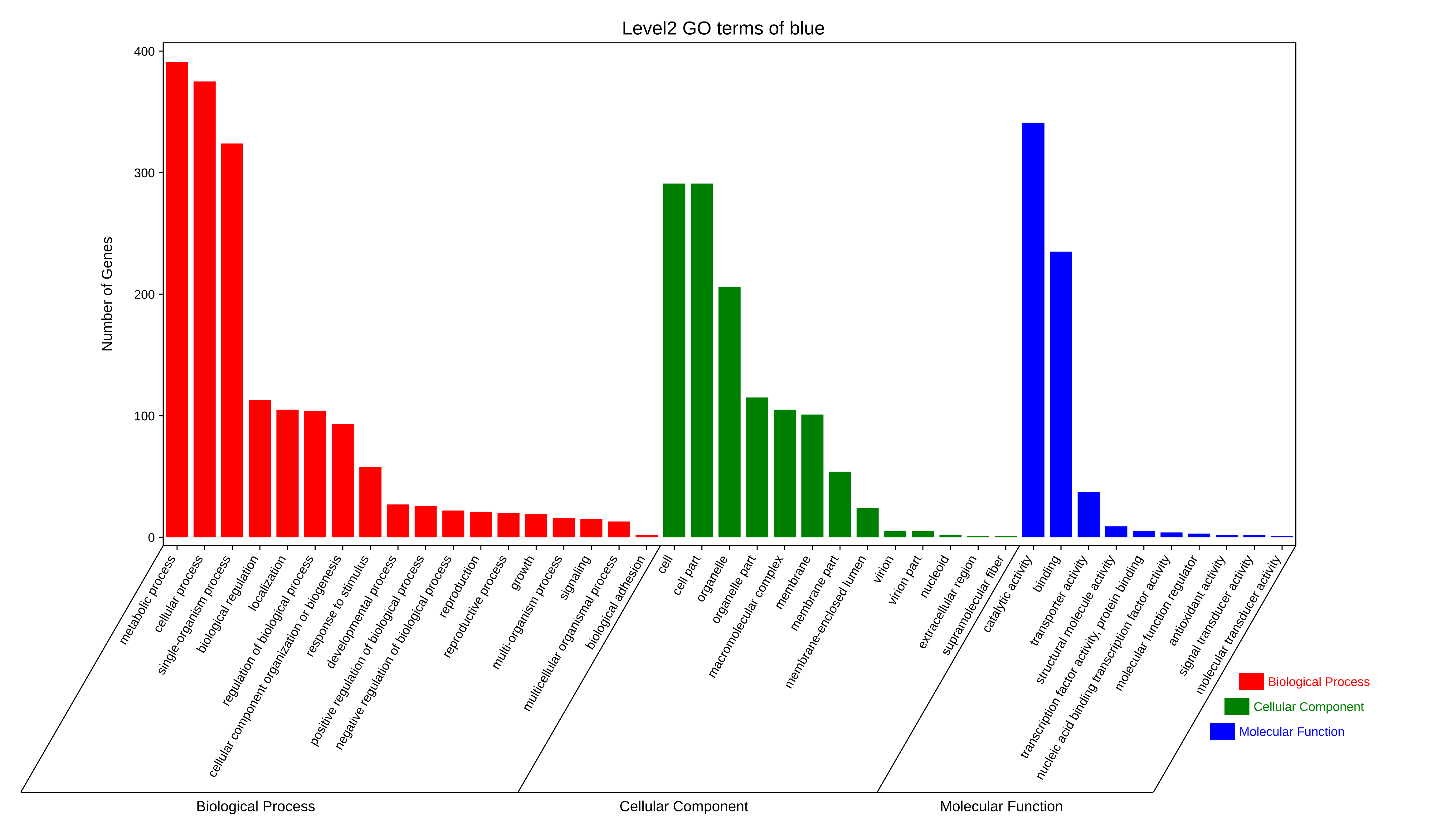


A


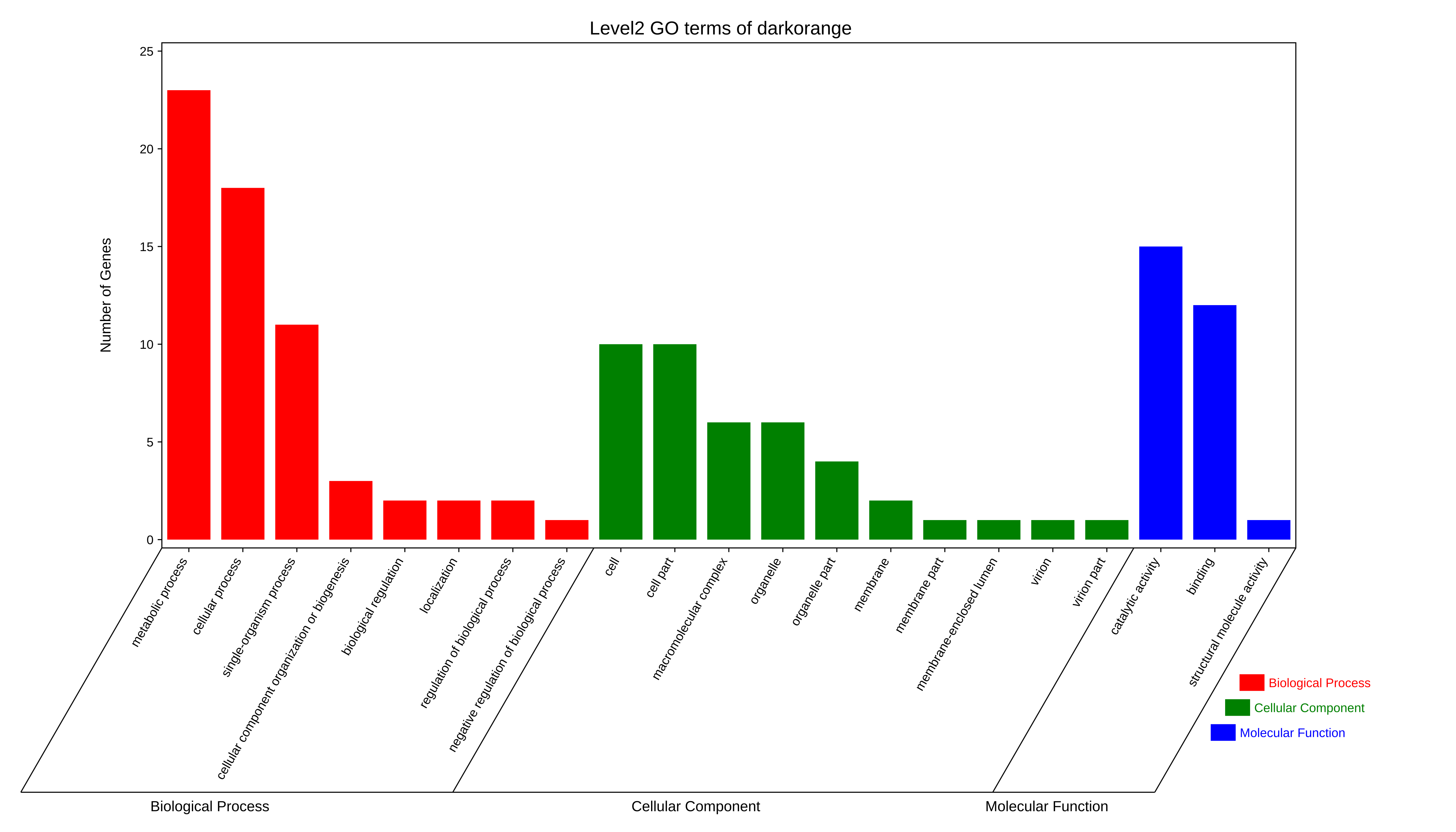


B


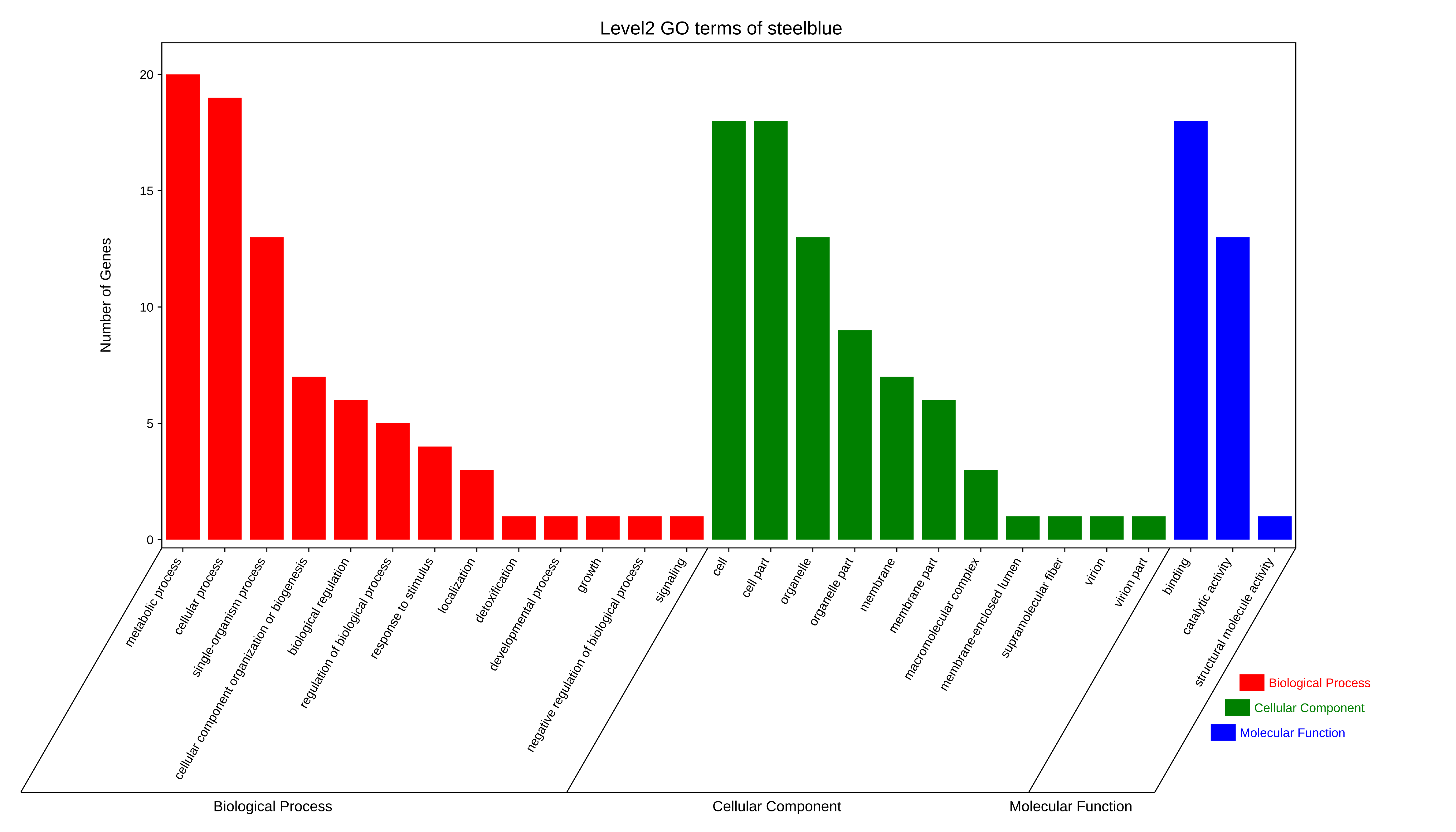


C


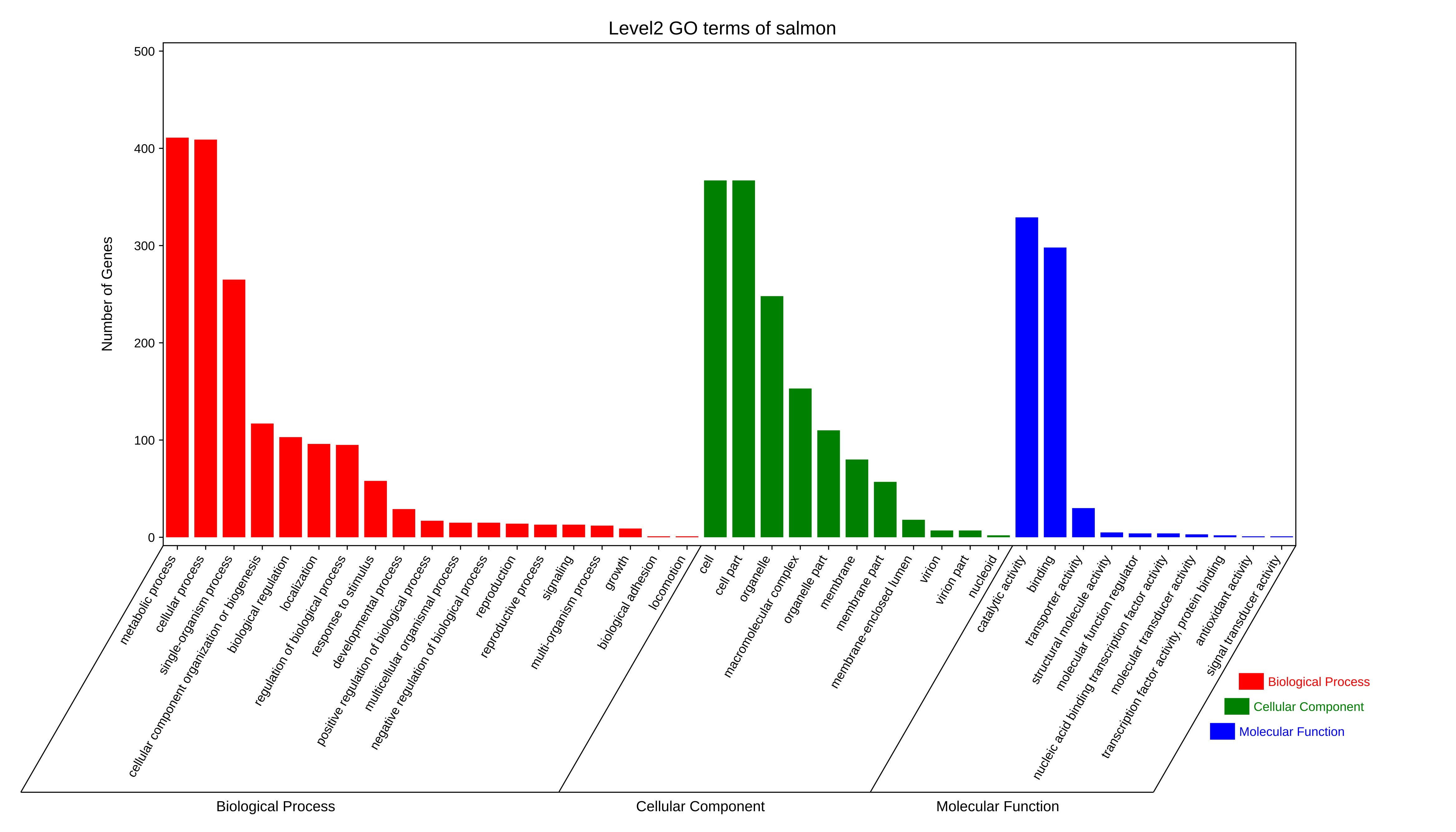


D

**Fig. S5.** Gene GO annotation in the target modules. **A**: blue module. **B**: darkorange module. **C**: steelblue module. **D**: salmon module.
