# Supplementary material for: Comparative transcriptome and WGCNA reveal key genes involved in lignocellulose degradation in Sarcomyxa edulis
Source: Sci Rep. 2022 Nov 1;12:18379. doi: 10.1038/s41598-022-23172-2 (PMC9626453; doi:10.1038/s41598-022-23172-2)
Supplement: Supplementary file 6 — Supplementary Information 6. [file 41598_2022_23172_MOESM6_ESM.doc]

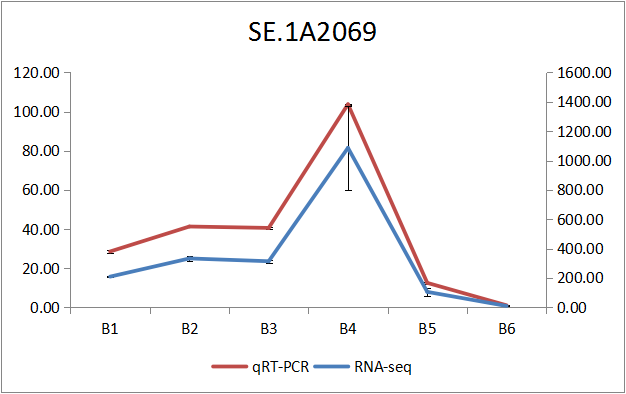

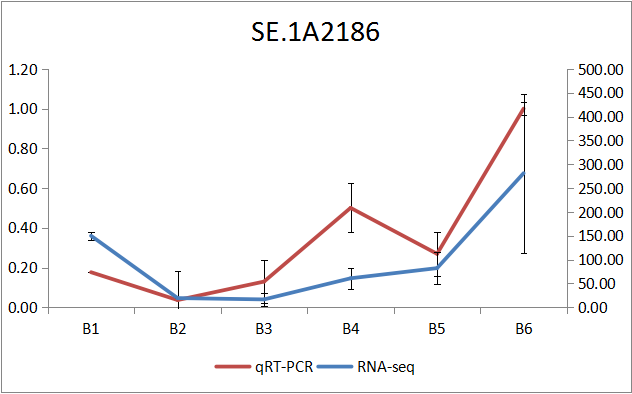


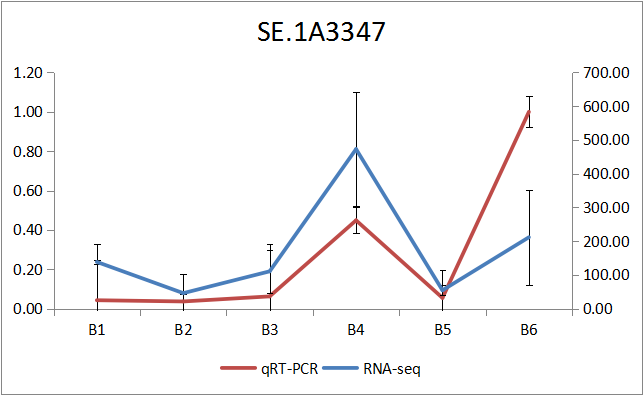

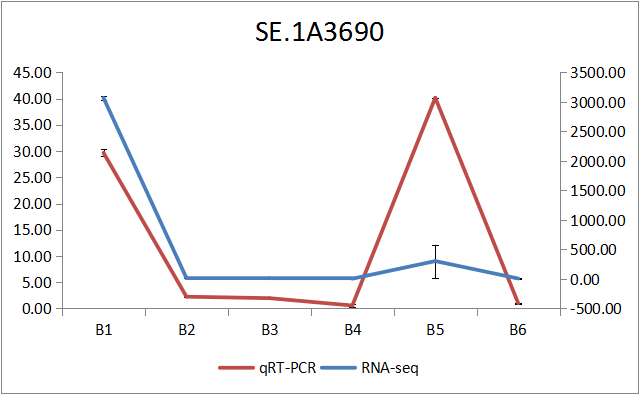


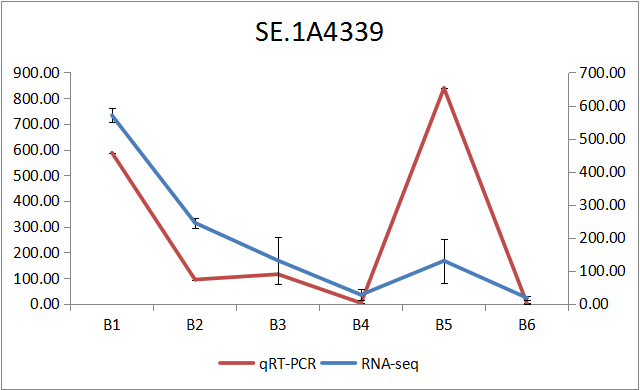

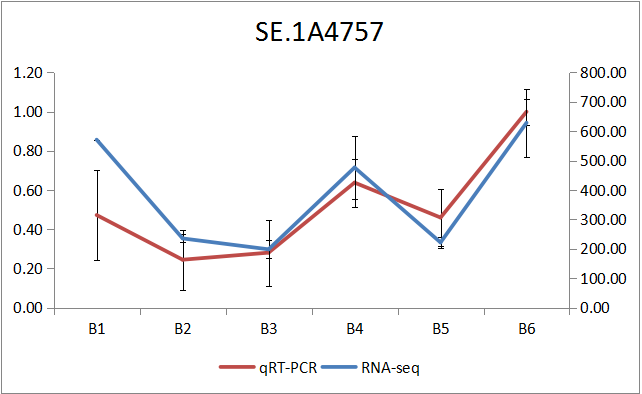


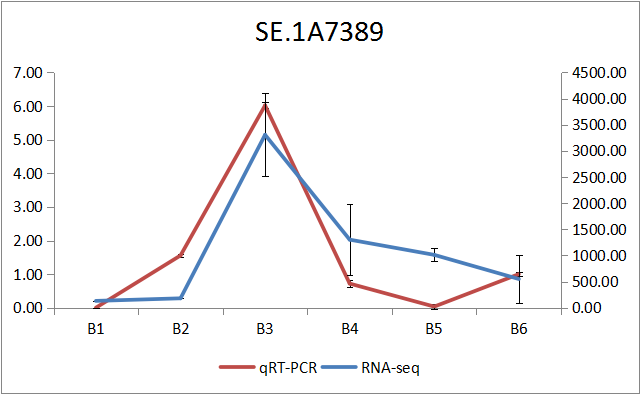

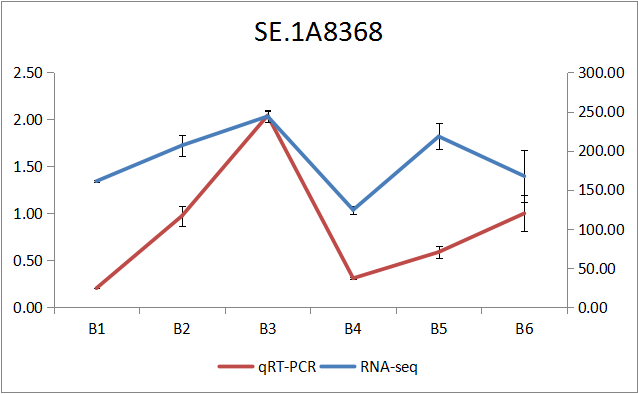


Fig. S6 The qRT-PCR analysis of gene expression compared to the RNA-seq data. The bars in the points represents the standard deviation.
