# Supplementary material for: Comparative transcriptome and WGCNA reveal key genes involved in lignocellulose degradation in Sarcomyxa edulis
Source: Sci Rep. 2022 Nov 1;12:18379. doi: 10.1038/s41598-022-23172-2 (PMC9626453; doi:10.1038/s41598-022-23172-2)
Supplement: Supplementary file 11 — Supplementary Information 11. [file 41598_2022_23172_MOESM11_ESM.doc]

| Gene number | Sequence of primers |
| --- | --- |
| ITS | F: 5' TCAGCAAAGACGGAGAAGGT 3'  R: 5' GATGAAGAACGCAGCGAAAT 3' |
| SE.1A2069 | F: 5' CCGAAGATGTACCCTGACCC 3'  R: 5' TCTCCCGAATCCAAACGC 3' |
| SE.1A2186 | F: 5' CTCACGGTGCTGATGGTAATG 3'  R: 5' TGTCGGAGATGAAGCTGGTC 3' |
| SE.1A3347 | F: 5' CTTGGTGGTTGGCTTGTCCT 3'  R: 5' TGCGAGTGGCTTTCGTCTT 3' |
| SE.1A3690 | F: 5' TAAACCAAGTCGGCAACAGC 3'  R: 5' TTGATCCCGCCAGCATAA 3' |
| SE.1A4339 | F : 5' AACGATAACGGAGGCGGAT 3'  R: 5' TGGTATTTGGGAACAATGCTG 3' |
| SE.1A4757 | F: 5' TGTCATTCCCACCGAGTTTG 3'  R: 5' AAAGTAGTAAGGAACCCAGTCAGC 3' |
| SE.1A7389 | F: 5' AACACGGGAACATCGGAGTC 3'  R: 5' GACACCTTCTAATTCGTTGCCTT 3' |
| SE.1A8368 | F: 5' AAAGTTCGGTTTCCAGAGGG 3'  R: 5' CTGAGCAGCGAAATACCACTT 3' |

Table S5 Primer sequences for qRT-PCR of the DEGs
